# Altered flexor carpi radialis motor axon excitability properties after cerebrovascular stroke

**DOI:** 10.3389/fneur.2023.1172960

**Published:** 2023-05-22

**Authors:** C.S. Klein, H. Liu, C. Zhao, W. Huang

**Affiliations:** Guangdong Work Injury Rehabilitation Center, Guangzhou, China

**Keywords:** stroke, nerve, ion channels, plasticity, flexor carpi radialis, threshold tracking, motoneuron, hyperexcitability

## Abstract

**Background:**

Spinal motoneurons may become hyperexcitable after a stroke. Knowledge about motoneuron hyperexcitability remains clinically important as it may contribute to a number of phenomena including spasticity, flexion synergies, and abnormal limb postures. Hyperexcitability seems to occur more often in muscles that flex the wrist and fingers (forearm flexors) compared to other upper limb muscles. The cause of hyperexcitability remains uncertain but may involve plastic changes in motoneurons and their axons.

**Aim:**

To characterize intrinsic membrane properties of flexor carpi radialis (FCR) motor axons after stroke using nerve excitability testing.

**Methods:**

Nerve excitability testing using threshold tracking techniques was applied to characterize FCR motor axon properties in persons who suffered a first-time unilateral cortical/subcortical stroke 23 to 308  days earlier. The median nerve was stimulated at the elbow bilaterally in 16 male stroke subjects (51.4 ± 2.9 y) with compound muscle action potentials recorded from the FCR. Nineteen age-matched males (52.7 ± 2.4 y) were also tested to serve as controls.

**Results:**

Axon parameters after stroke were consistent with bilateral hyperpolarization of the resting potential. Nonparetic and paretic side axons were modeled by a 2.6-fold increase in pump currents (IPumpNI) together with an increase (38%–33%) in internodal leak conductance (GLkI) and a decrease (23%–29%) in internodal H conductance (Ih) relative to control axons. A decrease (14%) in Na^+^ channel inactivation rate (Aah) was also needed to fit the paretic axon recovery cycle. “Fanning out” of threshold electrotonus and the resting I/V slope (stroke limbs combined) correlated with blood potassium [K^+^] (*R* = −0.61 to 0.62, *p*< 0.01) and disability (*R* = −0.58 to 0.55, *p* < 0.05), but not with spasticity, grip strength, or maximal FCR activity.

**Conclusion:**

In contrast to our expectations, FCR axons were not hyperexcitable after stroke. Rather, FCR axons were found to be hyperpolarized bilaterally post stroke, and this was associated with disability and [K^+^]. Reduced FCR axon excitability may represent a kind of bilateral trans-synaptic homeostatic mechanism that acts to minimize motoneuron hyperexcitability.

## 1. Introduction

Spinal motoneurons that innervate muscles of the paretic (contralesional) limb may become more excitable than normal after a cerebrovascular stroke (1). Laboratory measures of reflex or volitional electromyographic (EMG) activity indicate that paretic limb motoneuron hyperexcitability is prominent in forearm muscles that flex the wrist and fingers (“forearm flexors”) compared to other upper limb muscles; H-reflexes at intensities subliminal for motor axons are easily elicited in the forearm flexors but not the forearm extensors or intrinsic hand muscles (1); muscle stretch evokes above normal EMG amplitudes in the forearm flexors but not the thumb muscles (2, 3); during a voluntary isometric contraction, antagonist coactivation tends to be greater in forearm flexors than extensors (4); forearm flexor EMG activity may continue for several seconds after termination of a hand grip contraction in the paretic and nonparetic limb (5); during shoulder-loaded robot-assisted reaching tasks the EMG amplitude is larger in forearm flexors than extensors in the paretic and nonparetic limb relative to control (6). The findings indicating that the nonparetic forearm flexors are hyperexcitable during voluntary contractions is consistent with notion that this limb is also abnormal after stroke (7).

Knowledge about motoneuron hyperexcitability remains clinically important as it may influence the expression of a number of post-stroke phenomena, including spasticity, flexion synergies, and abnormal resting limb postures. The explanation for post-stroke predominance of forearm flexor hyperexcitability is uncertain. In theory, it may indicate that flexor motoneurons are prone to hyperexcitability because of differences in their intrinsic properties (i.e., resting membrane potential, ion conductance) (8) and/or synaptic inputs they receive (9). Verrier and colleagues found that most stroke subjects had above normal stretch-evoked flexor carpi radialis (FCR) EMG amplitude in the paretic limb that was accompanied by lower than normal spontaneous background EMG just prior to stretch (10). Relatively larger reflex EMG activity at relatively lower background EMG activity suggests that the motoneurons themselves are not intrinsically more excitable. In contrast, some investigators reported larger FCR H-reflex to maximal M-wave amplitudes (H/M ratio) in the paretic compared to the nonparetic limb or healthy control limb (11, 12), but others found no such increase (13). Hu and colleagues reported prolonged FCR motoneuron excitatory post-synaptic potentials (EPSP) in the paretic compared to the non-paretic limb post-stroke, estimated using a novel H-reflex protocol and model simulation (8). However, post-stroke changes in H-reflex responses may arise from altered central input (i.e., disinhibition) to the interneurons and motoneurons rather than altered excitability of the motoneurons themselves (14). The aforementioned studies, which relied on reflex or volitional EMG recordings, are limited in their ability to access intrinsic motoneuron properties independent of confounding effects of ongoing synaptic inputs.

Motoneuron and motor axon plasticity associated with pathological conditions may be linked. Thus, examination of axons may provide some insight into the etiology of motoneuron hyperexcitability. Nerve excitability testing using threshold tracking techniques can indirectly assess motor axon properties *in-vivo*, such as the resting membrane potential and ion channel conductance (15–19). Motor axons are complex structures made of functionally distinct domains, including nodes of Ranvier, paranodes, and internodes (20). These domains contain unique complements of ion channels, membrane pumps, and ion exchange processes, presumably to keep membrane potential and excitability (i.e., the propensity of an input to cause an action potential) within certain limits. The resting membrane potential is primarily determined by K^+^ ions, and secondarily Na^+^ ions, and the concentration differences of these ions across the cell membrane are maintained by the Na^+^/K^+^ pump (16). Indeed, some nerve excitability parameters have been found to be sensitive to blood serum potassium concentration [K^+^] (21, 22). Besides the passive flow of K^+^ and Na^+^ ions through their respective channels, other conductance active below action potential threshold, such as Ih (which flow through hyperpolarization-activated cyclic nucleotide channels-HCN), may also influence resting potential and excitability (23).

Motor axons, like motoneurons, that innervate different muscles have different excitability properties (24–26), even when they course through the same nerve (27–29). For example, threshold reductions during 100 ms subthreshold depolarizing currents are smaller, accommodation during strong 200 ms hyperpolarizing currents is less, and superexcitability is smaller, in FCR compared to abductor pollicis brevis (APB) axons (28). As these differences are evident at the same stimulus site (median nerve at the elbow), they may be explained mostly by differences in axon ion channel properties as opposed to axon architecture. The apparent muscle-dependent differences in ion channel properties are clinically relevant because they may influence axon plasticity associated with pathological conditions (30).

A stroke leads to plastic changes in motor axons. All post-stroke nerve excitability studies to date, except one, examined APB motor axons by stimulating the median nerve at the wrist and recording the APB compound muscle action potential (CMAP) (31–36). In one study of five stroke patients, FCR motor axon properties were examined to assess peripheral versus central effects of botox injection on spasticity, but data for healthy control FCR axons was not presented (37). With regard to the studies of APB axons, there was no strong evidence that axons were depolarized or more excitable after stroke. Rather, accommodation to membrane hyperpolarization was reduced in the paretic side axons, possibly due to lower Ih (31–35). The lack of an increase in APB axon excitability is consistent with the lack of augmented stretch reflexes in the thumb muscles after stroke (3), and together suggest that APB motoneurons and axons are not intrinsically more excitable after stroke. Whether this is also the case for the FCR is an open question. Axon plasticity may be muscle-dependent; post-stroke plasticity in APB axons may not necessarily represent plasticity in FCR axons, due in part to their apparent differences in ion channel properties (28).

The purpose of this study is to determine the differences in FCR motor axon excitability properties between the paretic and nonparetic limb in people after a stroke, and between the stroke limbs and the limb of healthy controls. We also determined whether any stroke-related differences in axon excitability properties are related to clinical features including disability, spasticity, maximal strength and EMG, and blood electrolytes. Based on previous reports of motoneuron hyperexcitability in the paretic forearm flexors post-stroke, we hypothesized that FCR axons would be similarly hyperexcitable, reflecting changes in ion channel conductance and/or the resting membrane potential. A report of some of these data was presented previously (38).

## 2. Materials and methods

### 2.1. Participants

We examined 16 males 23 to 308 days after suffering a first-time unilateral stroke (mean 100.2 ± 20.8 d, Table 1). They were hospital in-patients undergoing rehabilitation 5–6 days per week that included physiotherapy, occupational therapy, and traditional Chinese medicine treatments. Their mean age, height, and weight were 51.4 ± 2.9 y (range 30–72 y), 167.6 ± 1 cm, and 68.9 ± 2.2 kg. Nine had ischemic stroke and seven had hemorrhagic stroke. The unilateral lesion was located subcortically in 7, cortically in 4, and in both locations in 5, according to computer tomography or magnetic resonance images recorded at their acute care hospital. In all patients, routine blood work was done within 2 weeks on average of nerve testing (range 0–53 days). None had comorbidities (i.e., diabetes) or were taking medications that could impact peripheral nerve function or [K^+^] (i.e., diuretics). Nerve excitability was also recorded in 19 healthy aged-matched males who served as a control group. Their mean age, height, and weight were 52.7 ± 2.4 y (range 35–68 y), 168.2 ± 1.3 cm, and 66.2 ± 1.7 kg. Blood work was not done in the controls. Some of the controls participated in recreational sports once or twice per week, but none were highly trained. Informed written consent was obtained from all participants and all procedures were approved by the Guangdong Work Injury Rehabilitation Center Medical Ethics Committee (no. AF/SC-07/2015.28).

**Table 1:** Clinical information of the stroke participants.

ID	Age	WF MAS	EF MAS	Tendon reflex	HG MVC (kg)	TSO (days)	Paretic side	Lesion type	Lesion location
1	60	1	1	++	0	95	L	H	Basal ganglia
2	64	1+	1	+++	0	102	L	I	Temporal lobe
3	54	3	2	+++	0	46	L	H	Basal ganglia
4	56	1+	0	++	0	80	L	I	Frontal, temporal, parietal lobes
5	40	0	0	+	8.4	40	R	I	Basal ganglia
6	44	0	0	+	1.2	45	R	H	Basal ganglia
7	64	0	0	+	0	23	L	I	Temporal, parietal, occipital lobes, basal ganglia, external capsule
8	43	1+	1+	+++	4.7	107	R	H	Frontal, temporal parietal lobes, basal ganglia
9	50	1+	1+	+++	2.9	66	R	I	Frontal, temporal, occipital lobes, basal ganglia
10	64	1+	1+	+++	3.4	30	R	I	Basal ganglia
11	41	1	1+	+++	0	212	L	H	Temporal lobe
12	37	1+	1+	++	7.2	249	R	H	Temporal lobe
13	52	0	1	++	36	75	R	I	Temporal lobe, external capsule
14	72	1+	3	+++	0	82	R	H	Basal ganglia
15	52	1	1	+++	5.3	308	R	I	Frontal, temporal, parietal lobes, basal ganglia, internal capsule
16	30	1+	1+	+++	16.7	43	R	I	Pons

ID, identification number; WF MAS, wrist flexor Modified Ashworth score; EF MAS, elbow flexor Modified Ashworth score; Tendon reflex, deep tendon reflex (+ = decreased, ++ = normal, +++ = increased); HG MVC, hand grip maximal voluntary contraction force; TSO, time since onset of stroke; R, right side; L, left side; I, Ischemic; H, Hemorrhagic. The 16 participants are listed in order from lowest to highest total FIM score.

### 2.2. Assessment of disability and impairment

Disability level was determined by an occupational therapist using the Functional Independence measure (FIM). The FIM consists of 18 items (13 motor and 5 cognitive) rated on a 7-point ordinal scale that describes the level of independence. The severity of motor impairment was determined by a research physical therapist using the upper limb subscale of the Fugl-Meyer assessment (39). Spasticity of the wrist flexors, wrist extensors, and elbow flexors was determined according to the Modified Ashworth test, a 6-point scale that describes resistance to passive limb movement (40). The lowest score of 0 indicates no increase in muscle tone and the highest score of 4 indicates the affected part(s) are rigid in flexion or extension.

### 2.3. Maximal voluntary contraction (MVC) force and FCR EMG

Hand grip MVC force was determined using a dynamometer after nerve excitability testing was completed (Jamar Plus, Sammons Preston, Bolingbrook, IL). Three MVCs were recorded bilaterally in the stroke participants and unilaterally (right arm) in the controls. The MVC lasted about 4 s, with a 1 min rest period between each. FCR EMG during the MVC was recorded with the same electrodes used for nerve testing. The root-mean-square MVC EMG over a 2 s period was determined and divided by the FCR peak-to-peak CMAP. The highest MVC force and associated EMG were used in the calculation of group means.

### 2.4. Nerve excitability testing

Participants were seated with the shoulder abducted and flexed about 45°. The forearm rested on a padded table, with the elbow flexed about 110° (180° = full extension) and the wrist supinated. Elastic straps were placed across the forearm, palm, and fingers to minimize extraneous movements. In three stroke participants, paretic arm positioning was modified to accommodate abnormal limb postures. In two of these cases, the wrist was in neutral between supination and pronation (nos. 3 and 14). In one case, the forearm rested on a pillow in his lap with the elbow flexed 90° and the wrist pronated (no. 11).

Electrodes were positioned similar to the previous study of FCR axons in healthy adults (28). The median nerve was stimulated with a surface electrode (cathode) placed in the medial bicipital groove at the elbow. The electrode was lightly pressed into the groove by a padded plastic disc (4 cm diameter) and secured in place by an elastic strap that encircled the arm. The anode electrode was 10 cm proximally over the midline of the biceps brachii. The active EMG electrode was placed over the FCR, one-third the distance from the medial epicondyle to the radial styloid. The reference EMG electrode was placed over tendon at the wrist midline. A 1 cm square metal earth ground was placed on the lateral epicondyle. Silver-silver chloride electrodes (1 cm diameter snap button in a 2.2 cm × 2.2 cm adhesive cloth backing, Kendall H69P, Natus Neurology, WI, United States) were used for stimulation and EMG recording.

Stimulation and recording were controlled by QTracS software (© Prof. H. Bostock, Institute of Neurology, London). Pulses generated by computer were converted to current via a constant current stimulator (DS5, Digitimer Ltd., Welwyn Garden City, Hertfordshire, United Kingdom). EMG activity was amplified (×500), bandpass filtered (10 Hz to 3 kHz) (Astro-Medical, model P511, West Warwick, RI). Line frequency noise was removed on-line by a noise eliminator (Hum Bug 50/60 Hz Noise Eliminator, Digitimer Ltd). The EMG signal was digitized at a sampling rate of 10 kHz with a 16-bit converter (NI-USB6221; National Instruments; Austin, TX). The Trond protocol, consisting of five subroutines, was applied; stimulus response, strength-duration, threshold electrotonus, current-threshold, and recovery cycle properties (41).

Skin temperature, monitored by a thermistor close to the cathode (Omega Engineering Inc., Stamford, CT, United States), was kept at ≥32°C by covering the arm with towels. The probe was applied immediately as the participant was prepared for testing (i.e., informed consent, skin preparation etc.). Nerve testing commenced after about 10–15 min, when skin temperature had stabilized.

### 2.5. Nerve excitability analysis

The excitability parameters derived from the recordings were determined using the QtracP program. Onset latency was the time from stimulus artifact to half CMAP negative peak amplitude. The CMAP peak was the average of the last three responses of the stimulus–response curve.

The stimulus–response slope was calculated according to the following; stimulus eliciting a 75% peak response minus that evoking a 25% peak response, divided by that eliciting a 50% peak response. Rheobase was slope of the line of the stimulus width versus threshold charge plot, and the negative intercept of this line on the x-axis was the strength-duration time constant (SDTC). Superexcitability (%) was the minimum mean of three adjacent responses and subexcitability (%) was the maximum mean of three adjacent responses beyond 10 ms. Refractoriness was the threshold at the 2.5 ms conditioning-test pulse delay, and the relative refractory period (RRP) was the first intercept on the x-axis. The TEd (10–20 ms)% and TEd (90–100 ms)%, were the thresholds at the noted periods during depolarizing (+40%) stimuli and TEh (10–20 ms)% and TEh (90–100 ms)% were the corresponding thresholds during hyperpolarizing (−40%) stimuli. S2 accommodation was the difference in threshold (%) between the peak threshold and the threshold at 100 ms during the +40% current. The resting I/V slope was the slope of the threshold responses between −10 and + 10% currents. The minimal I/V slope was equal to the best fit straight line to each three adjacent points in turn. The hyperpolarizing slope was equal to the best fit straight line through the most hyperpolarized three points.

### 2.6. Mathematical modeling

Using the MEMFIT program (©Professor H. Bostock, Institute of Neurology, London), a mathematical model of the axon was applied to more fully interpret the differences in excitability properties between the limbs (42). The effects of changes in different parameters were determined on the goodness-of-fit of the model to the recorded thresholds. The excitability of the model nerve was tested relative to the recordings repeatedly to determine threshold with an accuracy of 0.5%. The “discrepancy” between the recordings and the model was obtained by weighting the errors of the four Trond components as follows: strength-duration data, 0.5; threshold electrotonus, 1; current-threshold, 1; and recovery cycle, 1. The model was run in unclamped mode to allow secondary changes in resting potential in response to changes in conductance or pump currents. Various optimization strategies were employed to best simulate the recordings, including determining the best change in each parameter, changing 1–3 parameters per pass, and repeated runs of two-parameter combinations.

The default parameters of the MEMFit program, as well as capacitance (node, myelin, and internode) and external K^+^ concentration were selected to be examined. The default parameters were nodal Na^+^ conductance (transient and persistent), K^+^ conductance (slow and fast at node and internode), internodal Ih conductance, leak conductance (node and internode), and the Barrett–Barrett conductance. In addition, activation rates of Na^+^ and K^+^ channels were also examined to best fit the recovery cycle of the paretic limb because changing conductance or capacitance proved to be less successful (i.e., without affecting other Trond components significantly).

### 2.7. Statistics

A paired *t*-test was used to characterize differences between the paretic and non-paretic limb. An unpaired *t*-test was applied to examine differences between the stroke limbs and the control limb. The relationship between different variables was determined with the Pearson correlation coefficient. Differences were considered statistically significant when *p* < 0.05, and data are presented as means ± SE.

## 3. Results

### 3.1. FCR motor axon excitability properties

**Figure 1:**
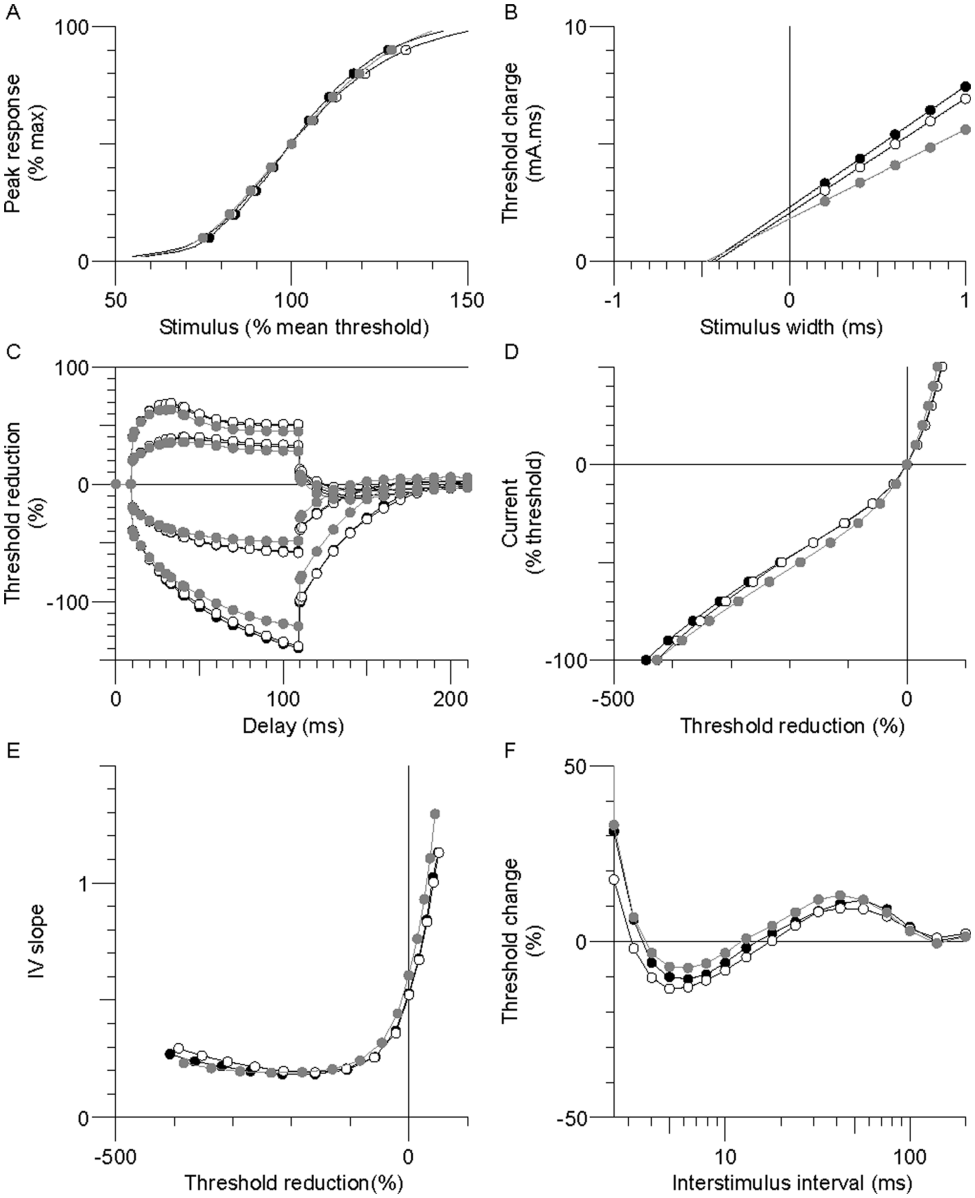
Group mean excitability profiles in the paretic (black symbols), nonparetic (white symbols), and control (grey symbols) limbs. **(A)** Normalized stimulus–response curve. **(B)** Threshold charge-stimulus width. **(C)** Threshold electrotonus. **(D)** Current-threshold (I/V). **(E)** I/V slope. **(F)** Recovery cycle.

Excitability recordings were completed bilaterally in the stroke participants and unilaterally in the controls. Skin temperature at the stimulus site was well controlled; paretic, nonparetic, and control means were not different (33.2 ± 0.2, 33.3 ± 0.2, and 33.0 ± 0.1°C, respectively, *p* > 0.2). Group mean axon responses are shown in a 6-plot format (Figure 1) and excitability parameter means are displayed in Table 2. In the following sections, axons of the paretic, nonparetic, and control limbs are sometimes referred to as “paretic axons,” “nonparetic axons,” and “control axons,” respectively.

**Table 2:** FCR axon excitability parameters in the paretic (P), non-paretic (NP), and control (C) limbs.

Excitability parameter	Paretic *n* = 16	Non-paretic *n* = 16	Control *n* = 19	P vs. NP *p*-value	P vs. C *p*-value	NP vs. C *p*-value
**Stimulus–response**						
CMAP peak (mV)	7.6 ± 0.7	9.4 ± 0.4	9.8 ± 0.3	0.01	0.006	0.4
Latency (ms)	5.0 ± 0.1	4.8 ± 0.1	4.7 ± 0.1	0.1	0.02	0.3
Stimulus (mA) for 50%max	7.9 ± 0.7	7.4 ± 0.6	5.9 ± 0.4	0.4	0.05	0.08
Stimulus–response slope	3.9 ± 0.2	3.4 ± 0.2	3.5 ± 0.1	0.01	0.1	0.8
**Stimulus width-charge**						
SDTC (ms)	0.456 ± 0.015	0.418 ± 0.016	0.487 ± 0.018	0.05	0.2	0.009
Rheobase (mA)	5.1 ± 0.5	4.9 ± 0.4	3.8 ± 0.3	0.6	0.06	0.07
**Recovery cycle**						
RRP (ms)	3.77 ± 0.21	3.23 ± 0.15	3.73 ± 0.16	0.0002	0.9	0.02
Refractoriness at 2.5 ms (%)	28.5 ± 5.4	17.6 ± 3.5	33.1 ± 3.6	0.0008	0.5	0.004
Superexcitability (%)	−10.5 ± 1.9	−12.9 ± 1.9	−8.2 ± 1.3	0.05	0.3	0.03
Subexcitability (%)	11.9 ± 1.8	10.2 ± 1.4	13.1 ± 1.6	0.4	0.6	0.2
**TE to ±40% currents**						
TEd (10–20 ms) (%)	65.6 ± 1.7	65.5 ± 1.7	61.9 ± 1.0	0.9	0.06	0.07
TEd (40–60 ms) (%)	54.7 ± 2.2	55.7 ± 2.0	49.7 ± 1.0	0.5	0.03	0.007
TEd (90–100 ms) (%)	50.4 ± 1.8	51.0 ± 1.6	44.9 ± 1.1	0.7	0.02	0.007
TEd (undershoot) (%)	−11.8 ± 0.7	−11.0 ± 1.0	−12.9 ± 0.6	0.6	0.3	0.1
S2 accommodation (%)	15.9 ± 0.6	16.0 ± 1.0	17.0 ± 0.7	0.8	0.2	0.4
Accommodation ½-time (ms)	41.5 ± 1.3	41.7 ± 0.7	40.2 ± 0.7	0.4	0.4	0.1
TEh (10–20 ms) (%)	−72.9 ± 1.9	−72.2 ± 1.4	−69.4 ± 1.1	0.6	0.1	0.1
TEh (20–40 ms) (%)	−93.7 ± 3.4	−91.9 ± 2.2	−85.8 ± 1.7	0.5	0.03	0.03
TEh (90–100 ms) (%)	−138.7 ± 7.1	−136.2 ± 5.5	−120.1 ± 3.5	0.6	0.01	0.01
TEh (overshoot) (%)	3.7 ± 0.8	3.7 ± 0.9	5.9 ± 0.6	0.5	0.04	0.07
**I/V relationship**						
Resting I/V slope	0.53 ± 0.03	0.52 ± 0.02	0.60 ± 0.01	0.6	0.02	0.009
Minimum I/V slope	0.17 ± 0.00	0.17 ± 0.00	0.18 ± 0.00	0.9	0.01	0.03
Hyperpolarizing I/V slope	0.27 ± 0.02	0.30 ± 0.02	0.23 ± 0.01	0.1	0.1	0.005

#### 3.1.1. Stimulus–response and strength-duration properties

**Figure 2:**
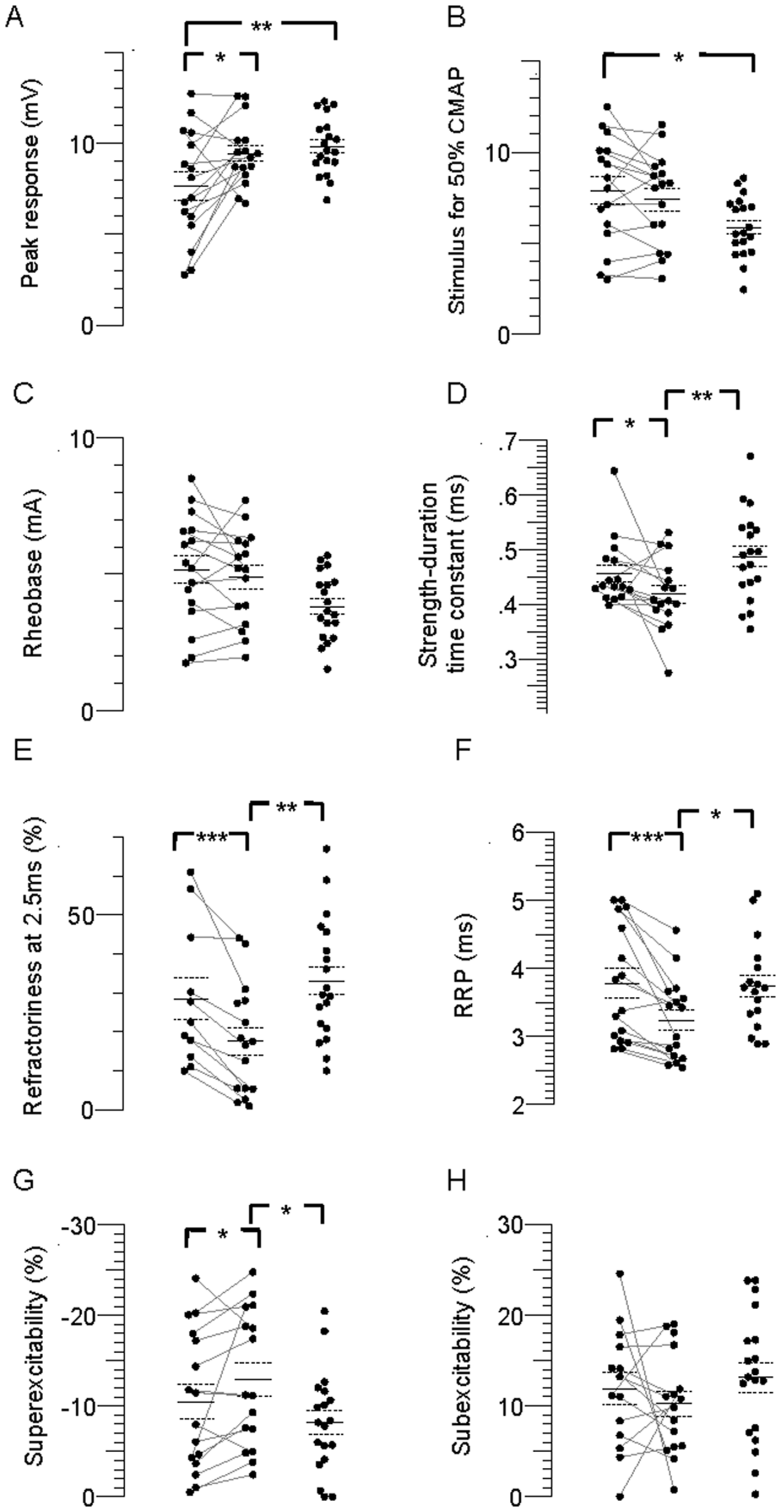
Individual stimulus–response, strength-duration, and recovery cycle parameters in the paretic, nonparetic, and control limbs. **(A)** CMAP peak amplitude. **(B)** Stimulus for 50% CMAP. **(C)** Rheobase. **(D)** SDTC. **(E)** Refractoriness. **(F)** RRP. **(G)** Superexcitability. **(H)** Subexcitability. The paretic (leftmost column of points) and non-paretic (middle column) limb for each person are represented by paired points connected by a line, and data for the control limb is displayed in the right column. The horizontal lines are group means and the dashed lines are standard errors. Significant difference between limbs; *p < 0.05, **p < 0.01, ***p < 0.001, ****p < 0.0001.

FCR CMAP peak amplitude was 20% and 23% smaller in the paretic compared to nonparetic and control axons, respectively (*p* = 0.01 and 0.006), whereas it was not different between nonparetic and control (*p* = 0.4, Figure 2A, Table 2). Stimulus currents for 50% CMAP and rheobase were not significantly different between the stroke limbs, whereas there was a trend for both parameters to be elevated compared to control (Figures 2B,C). The SDTC was longer in the paretic than the nonparetic axons (*p* = 0.05) but was not different between paretic and control (*p* = 0.2, Figure 2D). In contrast, SDTC was shorter in the nonparetic compared to control axons (*p* = 0.009).

#### 3.1.2. Recovery cycle

The recovery cycle differed between limbs, apparent during the refractory period. Refractoriness at the 2.5 ms delay was larger in the paretic than the nonparetic axons, whereas it was not different between paretic and control (Figure 2E, Table 2, *p* = 0.0008, paired *t*-test, *N* = 11). Note that refractoriness in the paretic limb was not recordable in 5 persons despite application of strong stimulus currents (threshold current for the 40% CMAP was not reached and the test was terminated by the investigator). The RRP was longer in the paretic compared to the nonparetic axons (*p* = 0.0002), whereas it was not different between paretic and control (Figure 2F). Mean refractoriness in the nonparetic limb for all 16 subjects (17.6 ± 3.5%) was not different compared to the subgroup of 11 subjects (17.2 ± 4.7%). Refractoriness and the RRP were smaller in the nonparetic than control axons (*p* = 0.004 and 0.02). Superexcitability was smaller in the paretic than nonparetic axons (*p* = 0.05), whereas it was not different between paretic and control (Figure 2G). In contrast, superexcitability was larger in the nonparetic than control axons (*p* = 0.03). Subexcitability was not different between any of the limbs (Figure 2H).

#### 3.1.3. Threshold electrotonus

Thresholds are lowered (i.e., increased excitability) during the application of prolonged (100 ms) subthreshold depolarizing conditioning currents, whereas the opposite occurs during hyperpolarizing currents. Threshold responses to either polarizing current were not different between the stroke limbs (Figure 1C, *p* > 0.05). However, threshold reductions were larger (or more “fanned out”) in both stroke limbs compared to control; i.e., reductions in threshold 90–100 ms after the start of the 40% current (TEd 90–100 ms) were about 11% larger, and the corresponding value for the −40% current (TEh 90–100 ms) was 15% (*p* < 0.03).

#### 3.1.4. Current-threshold (I/V) relationship

Limb differences in threshold behavior during 200 ms polarizing currents were consistent with threshold electrotonus; there were no differences in threshold between the stroke limbs during any polarizing currents (Figure 1D, *p* > 0.05). Mean threshold reductions were about 12%–20% larger in both stroke limbs during the 10%–50% currents, and 8%–28% larger during the −10% to −70% currents, compared to control (*p* < 0.05). There was greater steepening of the I/V plot in the stroke limbs during the −80% to −100% currents. As a result, the mean hyperpolarizing I/V slope was larger in both stroke limbs compared to control, although this was significant only in the nonparetic limb (Figure 1E, *p* = 0.005).

### 3.2. Modeling axon excitability

#### 3.2.1. Control limb

**Figure 3:**
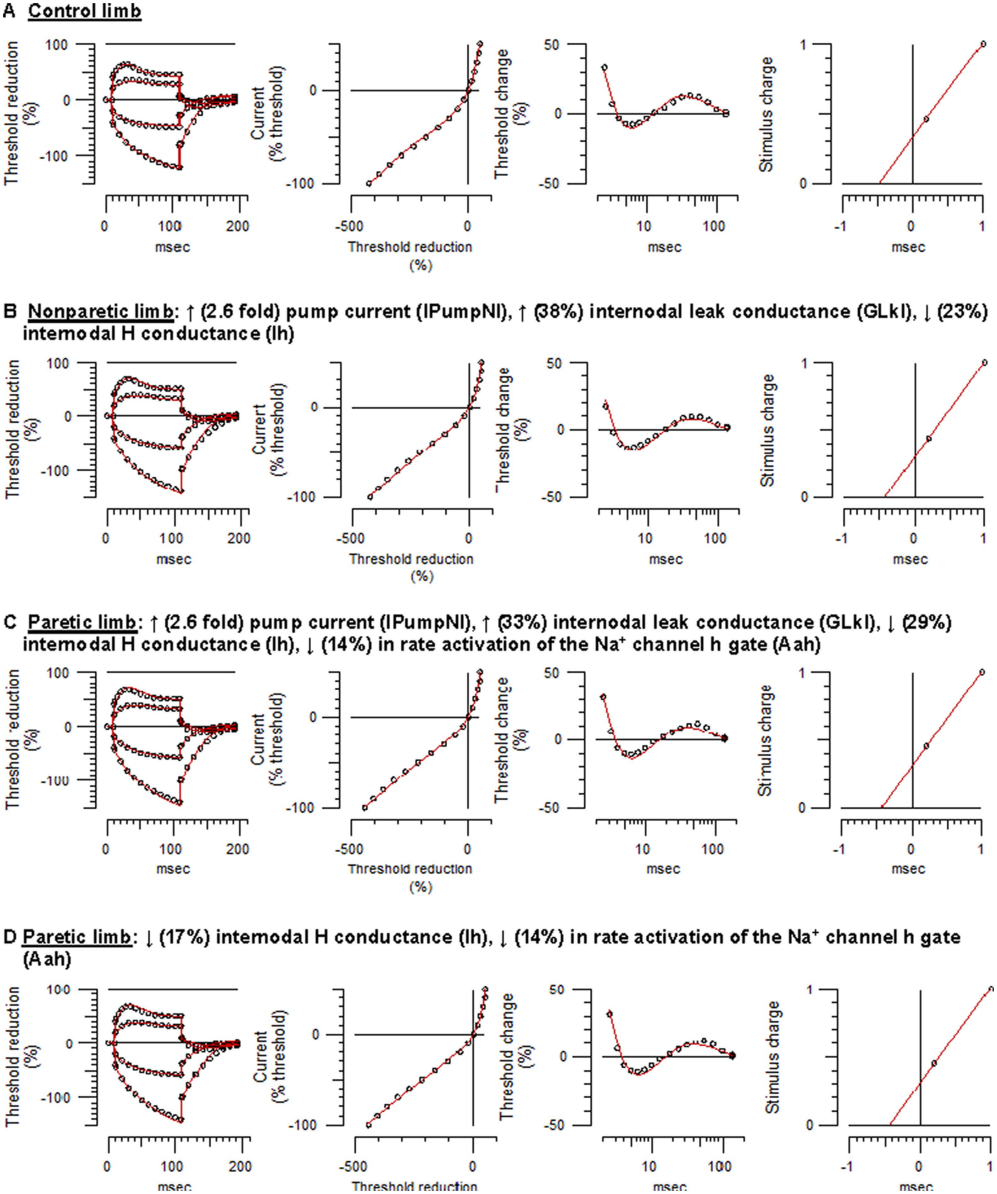
Mathematical modeling of FCR axonal behavior. Unfilled symbols are the recorded mean responses and the red traces are the best-fit models. The text above the panels are the relative changes in the modeled parameters that best simulated the nonparetic or paretic limb responses. **(A)** Control limb responses. **(B)** Nonparetic limb responses. **(C)** Paretic limb responses (modeling started with the control limb parameters). **(D)** Paretic limb responses (modeling started with the nonparetic limb parameters) (See Supplementary Figures S1–S4 and Supplementary Table S1 for further details).

Control limb responses were modeled first in order to examine membrane properties contributing to “normal” FCR axon behavior. A previously developed model characterizing APB axons at the wrist in healthy adults was used as a starting point (NC29 parameters included in the Qtrac software) (41). Compared to modeled APB axons, FCR axons had higher thresholds during strong hyperpolarizing currents of the I/V test, larger refractoriness, and smaller superexcitability (Supplementary Figure S1A), consistent with actual recorded differences between these axons in heathy adults (28). Changes in a number of the original APB axon model parameters was necessary to simulate FCR axon responses of the present study; reductions in Ih and the Barrett–Barrett conductance (GBB), as well as increases in nodal slow K^+^ conductance (GKsN) and internodal fast K^+^ conductance (GKfI), figured prominently (Figure 3A; Supplementary Table S1). The overall reduction in discrepancy between the recorded and modeled responses was 93.4%, reflecting 99.0%, 83.1%, 95.0%, and 95.3% discrepancy reductions in strength-duration, threshold electrotonus, current-threshold relationship, and recovery cycle, respectively.

#### 3.2.2. Nonparetic limb

Relative to control axons, the excitability profile of the nonparetic axons was consistent with hyperpolarization of the resting potential (43); larger fanning out of threshold electrotonus, lower resting I/V slope, shorter SDTC, and larger superexcitability. The following modeling results supports the notion that the resting potential was hyperpolarized after stroke. Starting with the modeled parameters for control FCR axons, the top four one-parameter changes that best fit the nonparetic recording were an increase in the pump currents (IPumpNI), increase in GBB, decrease in external K^+^ concentration (KO), and a decrease in Ih, where overall reductions in discrepancy were 83%, 50%, 46%, and 20%, respectively. After further repeated trials, optimizing different parameter combinations, nonparetic axons were best fit by a 2.6-fold increase in IPumpNI together with a modest increase (38%) in internodal leak conductance (GLkI) and a modest decrease (23%) in Ih (Figure 3B; see Supplementary Figure S2 for step-by-step details). The net result of these changes was a ~ 2.3 mV hyperpolarization of the resting potential. The overall reduction in discrepancy between the recording and the model was 87%, reflecting 99.3%, 84.5%, 86.6%, and 82.7% discrepancy reductions in strength-duration, threshold electrotonus, current-threshold relation, and recovery cycle, respectively.

#### 3.2.3. Paretic limb

Modeling suggested that paretic limb axons were also hyperpolarized relative to control axons, but contrary changes were also necessary to fit the recovery cycle. Starting with modeled parameters for the control axons, the top four one-parameter changes that best fit the paretic limb recording were an increase in IPumpNI, increase in GBB, decrease in KO, and a decrease in GLkI, where overall reductions in discrepancy were 77%, 48%, 36%, and 31%, respectively. After further optimization, paretic axons were best fit by a 2.6-fold increase in IPumpNI together with a modest increase (33%) in GLkI, a modest decrease (29%) in Ih, and a mild reduction (14%) in Na^+^ channel inactivation rate (Aah). The latter was necessary to best simulate the refractory period and superexcitability (Figure 3C; Supplementary Figure S3). The net result of these changes was a ~ 2.4 mV hyperpolarization of the resting potential. The overall reduction in discrepancy between the recording and the model was 80%, reflecting 44.7%, 84.5%, 57.5%, and 92.3% discrepancy reductions in strength-duration, threshold electrotonus, current-threshold relation, and recovery cycle, respectively.

Paretic axons were modeled again, but this time starting with the modeled parameters for the nonparetic limb. The results suggest subtle differences in ion channel properties between the stroke limbs; relative to nonparetic axons, paretic axons were best fit by a modest decreases in Ih and Aah (17% and 14%, respectively, Figure 3D; Supplementary Figure S4). The overall reduction in discrepancy between the recording and the model was 46%, reflecting 72.2%, 9.3%, 35.1% and 62.9% discrepancy reductions for the strength-duration, threshold electrotonus, current-threshold relation, and recovery cycle, respectively.

### 3.3. Disability and impairment

**Figure 4:**
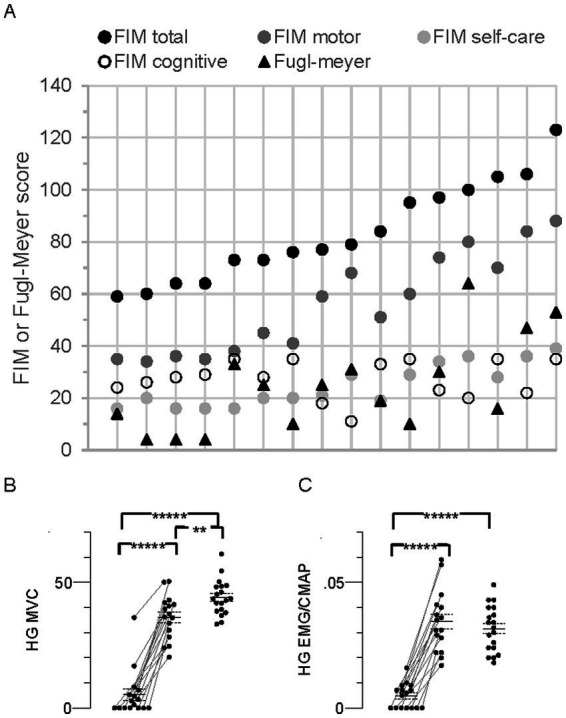
Individual Functional Independence Measure (FIM), Fugl-Meyer, and MVC in all stroke participants (*N* = 16). **(A)** Individual FIM and Fugl-Meyer scores. The data points for each person are arranged vertically and responses for the group are arranged from left to right according to FIM total score, consistent with the top-to-bottom arrangement in Table 1. FIM total score (maximum possible score = 126) together with the FIM subscale scores (motor, max = 91; self-care, max = 42; cognitive, max = 35), and Fugl-Meyer score (max = 66) are shown. **(B)** Hand grip (HG) maximal voluntary contraction (MVC) force (kg). **(C)** HG flexor carpi radialis (FCR) EMG amplitude normalized to the FCR compound muscle action potential (CMAP) peak-to-peak amplitude (HG EMG/CMAP). Data points in MVC panels arranged as described in Figure 2, Significant difference between limbs;*****p < 0.00001.

Disability according to FIM total score (equal to FIM motor + FIM cognitive subscale scores) ranged from moderate to mild (59 to 123, respectively, mean, 83.4 ± 4.7, Figure 4A). Total Fugl-Meyer scores, representing upper limb impairment, ranged from 4 to 64 (mean, 24.3 ± 4.5, Figure 4A). Spasticity was evident in 11 participants based on MAS ≥1 in both wrist flexors and elbow flexors, and tendon reflexes were augmented in 9 of these 11 (Table 1).

About one-third of the participants (nos. 1–5, Table 1) were characterized as “completely dependent” on assistance to carry out about half of the six FIM self-care tasks; i.e., individual ratings for each of bathing, dressing, and toileting were low (score of 1 or 2 out of 7), contributing to their low FIM self-care scores (16–20 out of a possible maximum score of 42, Figure 4A).

### 3.4. MVC force and FCR EMG

Seven persons with stroke were unable to generate any paretic limb grip force or EMG, suggesting complete paralysis of the FCR and other forearm muscles (Figures 4B,C). Furthermore, five of these seven also generated the lowest nonparetic MVC forces and EMG. Mean MVC force of the paretic limb was less than the nonparetic and control limbs by 85% and 88%, respectively (*p* = 1.9 × 10^−9^ and 4.9 × 10^−14^). Corresponding EMGs were similarly less by 86 and 85% (*p* = 1.5 × 10^−8^ and 3.8 × 10^−11^). Mean MVC force in the nonparetic limb was 18% less than control (*p* = 0.006), but there was no difference in the EMG (*p* = 0.4).

### 3.5. Correlations between excitability parameters and clinical measures

#### 3.5.1. [K^+^] and other blood serum constituents

**Figure 5:**
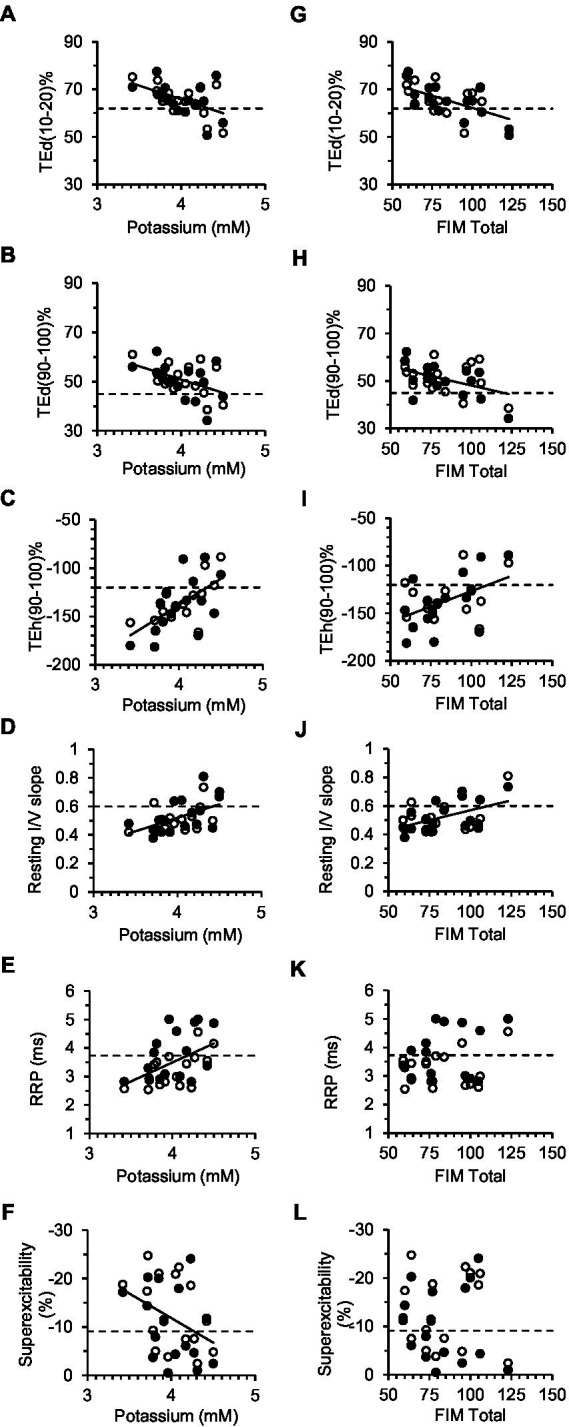
Correlations between excitability parameters, previously shown to be most sensitive to changes in the membrane potential, and serum [K^+^] **(A–F)** or FIM total **(G–L)**. Solid lines correspond to significant linear regressions between the excitability parameter (data of both limbs grouped together) and [K^+^] or FIM Total. Dashed lines are the control group means for the excitability parameters. In **A–F**, the intercept of the regression line with the dashed line provides an estimate of [K^+^] in a stroke subject with normal axon excitability (see Table 3 for corresponding equations and correlation coefficients).

Serum constituents that may impact excitability were, for the most part, normal in all stroke participants; [K^+^] ranged from 3.42 to 4.5 mmol/L (mean, 4.0 ± 0.07). Excitability parameters previously found to correlate best with current-induced changes in membrane potential in healthy adults (43) were found to be related mostly with [K^+^] in both stroke limbs. Thus, fanning out of threshold electrotonus and superexcitability were larger and RRP was shorter in individuals with lower [K^+^] (Figures 5A–F). A significant linear relationship between each of the six excitability parameters (data of both limbs grouped together) and recorded [K^+^] was obtained (Table 3; see Supplementary Tables S2, S3 for correlations in the paretic and nonparetic limbs separately). Based on these six regression equations, the [K^+^] of a stroke subject with a “normal” excitability parameter (i.e., equal to the control group mean indicated by dashed lines in Figure 5) was estimated to range from 4.17 to 4.55 mmol/L. The mean of these six estimates was 4.3 ± 0.05 mmol/L, which is about 7.5% higher than the 4.0 mmol/L actual recorded mean.

**Table 3:** Linear regression of FCR axon excitability parameters on blood serum [K^+^] after stroke.

	*R*	*P*	Regression equation	Estimated [K^+^] (mmol/L)
TEd (10–20 ms) %	−0.51	< 0.01	*y* = −11.6*x* + 112.3	4.34
TEd (90–100 ms) %	−0.47	<0.01	*y* = −10.8*x* + 94.1	4.55
TEh (90–100 ms) %	0.62	< 0.01	*y* = 54.2*x* − 354.9	4.33
Resting I/V slope	0.52	<0.01	*y* = 0.19*x* − 0.23	4.36
RRP (ms)	0.49	0.004	*y* = 1.33*x* − 1.83	4.17
Superexcitability %	0.39	0.028	*y* = 10.1*x* − 52.4	4.26

The correlation coefficients (*R*) and regression equations describe the best fit linear relationships between individual excitability parameters (data of both limbs grouped together) and individual recorded [K^+^]. Based on these regression equations, the estimated [K^+^] of a stroke subject with normal axon excitability (i.e., intercept of solid line and dashed line in Figure 5) is provided. *P*, probability of obtaining such a correlation by chance.

#### 3.5.2. Disability, impairment, and MVC

Disability was found to be related to some of the six excitability parameters previously found to be sensitive to the membrane potential (43). Specifically, fanning out of threshold electrotonus was larger, and resting I/V slope smaller, in persons with lower FIM total score; correlations (R) of FIM with TEd (10–20 ms)%, TEd (90–100 ms)%, TEh (90–100 ms)%, and resting I/V slope were −0.58 (*p* < 0.01), −0.44 (*p* < 0.05), 0.55 (*p* < 0.01), and 0.50 (*p* < 0.01), respectively (Figures 5G–J, data of both limbs grouped together). Excitability parameters did not correlate consistently with Fugl-Meyer, MAS, MVC, or stroke duration (data of both limbs grouped together).

## 4. Discussion

As stated in the introduction, clinical and laboratory evidence indicates that forearm flexor motoneurons of the paretic limb are often hyperexcitable following stroke. In addition, hyperexcitability seems to be prominent in the forearm flexors compared to the forearm extensors and intrinsic hand (thumb) muscles. Based on these previous findings, we hypothesized that paretic FCR axons would be hyperexcitable, but we found that this is not the case. Rather, the results suggest that FCR excitability is lower in both stroke limbs; the resting membrane potential was hyperpolarized bilaterally and Na^+^ channel inactivation rate was slowed in the paretic axons. The lack of an increase in axon excitability cannot be explained by atypical clinical features of the stroke participants. The clinical profile of the group was typical of unilateral moderate to severe cortical/subcortical lesions; paralysis, weakness (including in the nonparetic limb), wrist flexor spasticity, and inability to perform certain activities of daily living.

### 4.1. Altered FCR motor axon properties after stroke

#### 4.1.1. Bilateral hyperpolarization of the resting membrane potential

Axon excitability properties characterized by the Trond protocol are sensitive to the resting membrane potential. Thus, when the resting potential is hyperpolarized by passing background small DC hyperpolarizing currents through the stimulating electrodes, the axon excitability profile is altered; threshold electrotonus is fanned out, resting I/V slope is reduced, SDTC is shortened, and the recovery cycle is shifted downwards resulting in a shortened RRP and larger superexcitability (43). The nonparetic axon excitability profile and modeling are consistent with hyperpolarization of the resting potential relative to the control axons (44). Some, but not all, of the paretic axon excitability profile is also consistent with hyperpolarization compared to control axons. Thus, there was fanning out of threshold electrotonus and reduced resting I/V slope, but no downward shift in the recovery cycle nor shortened SDTC. Based on modeling, we propose that slowing in the rate of Na^+^ channel inactivation counteracted the downward shift in the recovery cycle associated with membrane hyperpolarization (see also Section 4.1.3).

We found no strong evidence that persistent Na^+^ or K^+^ were altered in the stroke axons, so they are less likely to directly account for the hyperpolarized resting potential. Similarly, only 0.2 mV of the 2.3 mV hyperpolarization was explained by the combined lower Ih and higher GLkI after stroke. Most of the hyperpolarization (2.1 mV) was explained by a 2.6 fold increase in “pump” currents, presumably reflecting increased Na^+^/K^+^ATPase activity. The Na^+^/K^+^ pump is normally hyperpolarizing, pumping 3 Na^+^ ions out of the cell for every 2 K^+^ ions imported, thereby helping to maintain ionic balance and excitability (45).

#### 4.1.2. Bilateral changes in HCN channel properties

The stroke-control group differences in threshold behavior during prolonged hyperpolarizing stimuli could not be explained entirely by differences in the resting potential. It was also necessary to decrease Ih and increase GLkI to best simulate hyperpolarizing threshold electrotonus and I/V responses in the stroke axons. In mammals, there are four HCN channel isoforms (HCN1-4) that differ in kinetics, voltage dependence, and sensitivity to cAMP (46, 47). For example, HCN1 is activated at more depolarized potentials and has a faster activation speed compared to HCN2-4, with HCN4 activated at the most hyperpolarized potentials and at the slowest speeds. A portion of GLkI may reflect slower HCN isoforms that are otherwise unmodeled (23). Thus, increased expression of slow at the expense of fast HCN isoforms may partially account for the larger bilateral threshold reductions over the first 200 ms of the hyperpolarizing currents.

There was greater steepening of the I/V relationship during the strongest hyperpolarizing currents in stroke compared to control axons, more clearly shown by the higher hyperpolarizing I/V slope in the former than the latter (Figure 1E, significant only in the nonparetic limb). This cannot be explained by more HCN channels because Ih was less than control (Supplementary Table S1). Rather, greater steepening may be explained by larger current flow through HCN channels due to greater resting membrane hyperpolarization (23).

#### 4.1.3. Slowing of Na^+^ channel inactivation in the paretic axons

Axons are less excitable for a few milliseconds after the passage of an impulse and is referred to as the refractory period. Refractoriness and the RRP are related to how quickly nodal Na^+^ channels recover from inactivation. Both refractory parameters were larger in the paretic than the nonparetic limb and modeling indicated that the cause was a lower value for the *h* gating particle (the particle that controls inactivation), or in other words a slower inactivation rate of Na^+^ channels. Slower inactivation rate of Na^+^ channels in the paretic axons suggests that relatively fewer Na^+^ channels are in the open state to generate impulses during the RRP.

### 4.2. Axon plasticity after stroke

Why and how changes in motor axon properties occur after a stroke is not well understood. It has been proposed that a stroke results in a net increase in tonic bilateral descending excitatory (monoaminergic) drive from central structures including the brain stem and propriospinal neurons (14, 48–51). Excessive descending excitatory inputs, possibly arising from disinhibited central structures, may be a key determinant of spasticity and other types of muscle “overactivity,” particularly in the forearm flexors. Reduced FCR axon excitability may represent a kind of trans-synaptic homeostatic mechanism to help minimize spasticity arising from increased descending excitatory inputs to the spinal motoneurons (however, see Section 4.2.2).

Our findings raise an interesting question; whether qualitatively similar plastic changes occur in FCR motoneurons after stroke. Thus, although the propensity for afferent inputs to recruit multiple motor units may be above normal, due to excessive tonic excitatory drive, a hyperpolarized resting potential may lessen this propensity. In other words, if not for the reduction in intrinsic axon (motoneuron) excitability, spasticity may have been more severe. Specifically, a hyperpolarized resting potential would tend to keep the membrane potential below threshold for activation of persistent inward currents and self-sustained firing (52). Also, if one assumes that spontaneous repetitive motoneuron (axon) discharges are more likely to occur in the paretic limb due to excessive excitatory inputs (50, 53), then slowed Na^+^ channel inactivation may be a way to minimize their occurrence. It is noteworthy that serotonin (which presumably is increased after stroke due to greater influence of monoaminergic brain stem pathways) can have inhibitory effects in isolated motoneurons (i.e., cause membrane hyperpolarization and decreased firing) in addition to the often described excitatory effects (54, 55).

#### 4.2.1. Axon plasticity and deficits in bilateral function

Either brain hemisphere has the capacity to influence axon plasticity bilaterally due in part to extensive bilateral descending projections emanating from the brain stem and other subcortical structures (56). The association of weakness between the stroke limbs (i.e., those with the lowest MVC in the paretic limb also had the lowest MVC in the nonparetic limb) is consistent with a unilateral stroke lesion affecting bilateral tracts (Figure 4B). Furthermore, the degree of fanning out of threshold electrotonus varied significantly with the level of disability (FIM) (Figures 5G–I), but not with MVC force, MVC EMG, or MAS. Indeed, deficits in FCR MVC EMG were severe in the paretic limb but were not evident in the nonparetic limb (Figure 4C), despite similar hyperpolarization of the resting potential. Thus, activation capacity, and presumably residual daily axon impulse traffic, may not be primary determinants of bilateral axon plasticity revealed here. The correlation of fanning out of threshold electrotonus and FIM may indicate that some of the post-stroke axon plasticity is related more to deficits in bilateral central nervous system processing (i.e., reduced ability to use compensatory strategies to complete certain FIM tasks) rather than deficits in maximal activation (7, 57, 58). Interestingly, fanning out in APB axons was associated with disability after severe acute cerebellar stroke (32) but not after cortical or subcortical stroke (33). APB axon excitability is also reduced in persons with cerebral palsy and multiple sclerosis (59, 60), although the underlying changes in ion channel properties seem to differ compared to FCR axons after stroke.

Of the many bilateral tracts that could influence axon plasticity, the corticospinal tract may have the least impact as only about 10% of the fibers are uncrossed (56, 61). Stronger candidates are the reticulospinal or medial vestibulospinal tracts as both have significant numbers of fibers that project bilaterally in the cervical cord (56). Reticulospinal inputs to the forearm flexor and intrinsic hand motoneurons are similarly strengthened subsequent to corticospinal lesions, whereas inputs to the forearm extensor motoneurons are not strengthened (61). Thus, the apparent differences in post-stroke responses of FCR versus APB axons may be unrelated to reticulospinal drive (see Section 4.3.2).

#### 4.2.2. Axon plasticity and serum potassium [K^+^]

The stroke FCR axons may be hyperpolarized because of lower [K^+^] as opposed to arising from altered spinal synaptic inputs. Excitability parameters after stroke were associated with [K^+^], consistent with previous control and patient data (21, 22, 62). Although hypokalemia ([K^+^] < 3.5 mmol/L) was evident in only one person, a 7.5% reduction in group mean [K^+^] could, in theory, explain membrane hyperpolarization. Indeed, the mean recorded [K^+^] in the stroke group (4.0 mmol/L) is about 7%–10% below normal values for Chinese adults (4.3–4.4 mmol/L) (63, 64), although similar to means of people in the United States (4.0 mmol/L) (65). In contrast, results in some individuals are less supportive of the notion that lower [K^+^] caused membrane hyperpolarization. For instance, fanning out of threshold electrotonus in the paretic axons of two persons is at or beyond the 95% confidence interval for the controls, despite apparently normal [K^+^] (4.42 and 4.42 mmol/L, no. 1 and no. 14, respectively, Figures 5A–D). Also, in a previous study of healthy adults, serial measurements over a 2 week period revealed no relationship between individual fluctuations in [K^+^] (more than 20% in some cases) and individual fluctuations in APB axon excitability parameters (superexcitability, RRP) (62). We can conclude that our data shows that [K^+^] and axon excitability are also associated in people who suffered a stroke. However, to clarify the influence of [K^+^] on post-stroke axon plasticity, serial recordings of both measures starting in the acute phase are necessary.

### 4.3. Comparison of FCR and APB excitability properties

#### 4.3.1. Modeled differences between healthy APB and FCR axons

Differences in Trond excitability properties between healthy control APB and FCR axons were previously reported, but modeling was not done to more fully interpret the differences (28). Our modeling quantified differences in ion channel properties between FCR and APB axons (the original APB model parameters) that may contribute to differences in their behavior (Supplementary Table S1). Specifically, lower GBB (i.e., passive discharge under the myelin sheath) and higher K^+^ conductance (GKsN, GKfI) may explain smaller depolarizing threshold electrotonus and superexcitability, and lower Ih may account for much of the smaller accommodation to hyperpolarizing stimuli, in FCR compared to APB axons.

#### 4.3.2. Post-stroke axon plasticity may be muscle-dependent

The stroke-induced FCR axon plasticity shown here appears to differ from APB axon plasticity reported previously. The most conspicuous difference is the lack of strong evidence for a change in the resting potential of APB axons after stroke (33, 34). Another difference is accommodation during hyperpolarizing currents is normal, or may be above normal, in nonparetic APB axons (34, 35) whereas it is less in FCR axons after stroke. These apparent different responses between APB and FCR axons after stroke raise the possibility that inherent differences in their ion channel properties, in addition to differences in synaptic inputs, may influence their plasticity. This notion needs to be confirmed by concurrent longitudinal recordings of APB and FCR axon excitability after stroke. We can confirm, however, that our control FCR parameter means are similar to published control data (Supplementary Table S4) (28), and our stroke FCR parameter means compare favorably with the previously reported FCR data in five chronic stroke subjects (Supplementary Table S5) (37).

## 5. Conclusion

Our findings further highlight the utility of recording a full excitability profile consisting of multiple measures of excitability (41). Thus, we would be unable to conclude with confidence that stroke axons are hyperpolarized bilaterally if only the recovery cycle was recorded, nor that Na^+^ channel gating is altered in paretic axons if only responses to subthreshold polarizing currents were recorded. The post-stroke axon plasticity revealed here may be triggered by excessive descending excitatory inputs, although a contribution from lower serum [K^+^] cannot be ruled out.

## Data availability statement

The original contributions presented in the study are included in the article/Supplementary material, further inquiries can be directed to the corresponding author.

## Ethics statement

The studies involving human participants were reviewed and approved by Medical Ethics Committee of the Guangdong Work Injury Rehabilitation Center. The patients/participants provided their written informed consent to participate in this study.

## Author contributions

CSK, HL, CZ, and WH conceived the study. WH helped with recruitment and provided all medical support for the stroke participants. CSK, HL, and CZ collected the data. HL and CZ conducted physical assessments of the patients. CSK analyzed the data and wrote manuscript drafts. All authors contributed to the article and approved the submitted version.

## Funding

This study was supported by the Guangzhou Science and Technology Program key projects: Number 201904010256.

## Conflict of interest

The authors declare that the research was conducted in the absence of any commercial or financial relationships that could be construed as a potential conflict of interest.

## Publisher’s note

All claims expressed in this article are solely those of the authors and do not necessarily represent those of their affiliated organizations, or those of the publisher, the editors and the reviewers. Any product that may be evaluated in this article, or claim that may be made by its manufacturer, is not guaranteed or endorsed by the publisher.
