# Supplementary material for: Altered flexor carpi radialis motor axon excitability properties after cerebrovascular stroke
Source: Front Neurol. 2023 May 22;14:1172960. doi: 10.3389/fneur.2023.1172960 (PMC10240235; doi:10.3389/fneur.2023.1172960)
Supplement: Supplementary file 1 [file Data_Sheet_1.PDF]

Table 1. Final model parameters that best fit healthy control, nonparetic, and paretic FCR axons

Parameter	Original model	Control limb	Nonparetic limb	Paretic limb
ENR<br>Nodal resting potential	-82.7	-85.5	**-87.8**	**-87.8**
EIR<br>Internodal resting potential	-82.7	-85.7	**-88.1**	**-88.2**
PNaN<br>Nodal Na^+ permeability	4.1	4.1	4.1	4.1
PNap(%)<br>Percent persistent sodium	0.895	0.895	0.895	0.895
GKsN<br>Nodal slow K^+ conductance	47.6	54	54	54
GKsI<br>Internodal slow K^+ conductance	0.24	0.24	0.24	0.24
GKfN<br>Nodal fast K+ conductance	22	20	20	20
GKfI<br>Internodal fast K+ conductance	100	175	175	175
Ih<br>Internodal H conductance	6.3	1.55	**1.2**	**1.1**
GLkN<br>Nodal leak conductance	1.53	1.53	1.53	1.53
GLkI<br>Internodal leak conductance	2.05	2.25	**3.1**	**3.0**
GBB<br>Barrett-Barrett conductance	35.8	27.7	27.7	27.7
CMy<br>Myelin capacitance	1.55	2.2	2.2	2.2
CAX<br>Internodal capacitance	0.273	0.253	0.253	0.253
IPumpNI<br>Nodal and intermodal pump currents	0	0.0068	**0.0175**	**0.0175**
Aah<br>Rate activation of Na+ channel h gate	0.0245	0.0245	0.0245	**0.021**
Tabs<br>Absolute temperature	301.8	302.5	302.5	302.5

Axon parameters of the original model, and FCR parameters in the control, nonparetic, and paretic limb.The stroke limb parameters that differed from the control limb parameters are bolded in red. The original model parameters correspond to the best-fit simulation of APB axon responses in healthy adults described previously (Kiernan et al., 2000) (NC29 parameters included in the Qtrac software).

Table 2. Means of blood serum constituents and correlations of these constituents with selected FCR axon excitability parameters in the paretic limb after stroke.

	Mean ± SE range	TEd(10-20)%		TEd(90-100)%		TEh(90-100)%		Resting I/V slope		RRP		Superexcitability	
		R	*P*	*R*	*P*	*R*	*P*	*R*	*P*	*R*	*P*	*R*	*P*
Potassium (3.5-5)	4.0 ± 0.07 3.42-4.5	-0.42	0.10	-0.47	0.06	**0.59**	**0.01**	**0.51**	**0.04**	**0.48**	**0.05**	0.38	0.17
Sodium (135-147)	143.4 ± 0.5 140-147	-0.03	0.8	0.05	0.82	-0.12	0.66	0.11	0.68	0.03	0.88	0.11	0.67
Chloride (96-106)	103.1 ± 0.6 97.4-108	-0.13	0.63	-0.13	0.61	0.15	0.57	0.18	0.50	-0.11	0.67	-0.19	0.48
Calcium (2.0-2.5)	2.28 ± 0.02 2,13-2.42	**-0.52**	**0.04**	-0.48	0.07	**0.56**	**0.03**	0.32	0.23	0.23	0.41	0.46	0.08
Magnesium (0.8-1.25)	1.0 ± 0.03 0.84-1.26	0.47	0.07	**0.55**	**0.03**	-0.32	0.24	-0.39	0.14	-0.47	0.07	-0.43	0.10
Glucose (3.89-6.11)	5.14 ± 0.16 4.31-7.02	-0.07	0.79	0.02	0.89	-0.02	0.88	-0.11	0.67	0.13	0.63	0.25	0.35
Urea (1.7-8.3)	4.32 ± 0.31 2.83-6.86	-0.34	0.21	-0.10	0.70	0.28	0.31	0.30	0.27	0.27	0.33	0.24	0.38
Creatine (40-124)	75.8 ± 3.5 57-111	-0.27	0.32	-0.09	0.72	0.10	0.70	-0.13	0.63	-0.15	0.60	-0.03	0.88

Serum levels are expressed in mmol/L except for creatine (μmol/L). Normal ranges for serum concentrations are shown in brackets. *R* = the correlation coefficient between the excitability parameter and the serum concentration. *P* = the probability of obtaining such a correlation by chance. Significant correlations are bolded.

Table 3. Means of blood serum constituents and correlations of these constituents with selected FCR axon excitability parameters in the nonparetic limb after stroke.

	Mean ± SE range	TEd(10-20)%		TEd(90-100)%		TEh(90-100)%		Resting I/V slope		RRP		Superexcitability	
		R	*P*	*R*	*P*	*R*	*P*	*R*	*P*	*R*	*P*	*R*	*P*
Potassium (3.5-5)	4.0 ± 0.07 3.42-4.5	**-0.59**	**0.01**	**-0.48**	**0.05**	**0.68**	**0.003**	**0.54**	**0.03**	**0.61**	**0.01**	0.43	0.09
Sodium (135-147)	143.4 ± 0.5 140-147	0.18	0.50	0.30	0.25	-0.17	0.52	-0.30	0.21	-0.23	0.39	0.17	0.52
Chloride (96-106)	103.1 ± 0.6 97.4-108	-0.06	0.81	0.02	0.90	0.34	0.18	0.21	0.42	-0.11	0.67	-0.02	0.88
Calcium (2.0-2.5)	2.28 ± 0.02 2,13-2.42	-0.42	0.10	-0.42	0.11	0.44	0.09	0.23	0.42	0.46	0.07	0.42	0.11
Magnesium (0.8-1.25)	1.0 ± 0.03 0.84-1.26	0.49	0.06	**0.64**	**0.01**	-0.22	0.43	**-0.51**	**0.05**	-0.40	0.13	-0.44	0.09
Glucose (3.89-6.11)	5.14 ± 0.16 4.31-7.02	-0.25	0.35	-0.31	0.24	-0.10	0.69	0.07	0.77	0.09	0.71	0.27	0.29
Urea (1.7-8.3)	4.32 ± 0.31 2.83-6.86	-0.15	0.58	0.13	0.65	0.08	0.77	-0.30	0.27	0.01	0.90	-0.02	0.90
Creatine (40-124)	75.8 ± 3.5 57-111	0.31	0.26	-0.02	0.88	-0.09	0.73	-0.24	0.38	-0.23	0.41	0.05	0.84

Serum levels are expressed in mmol/L except for creatine (μmol/L). Normal ranges for serum concentrations are shown in brackets. *R* = the correlation coefficient between the excitability parameter and the serum concentration. *P* = the probability of obtaining such a correlation by chance. Significant correlations are bolded.

Table 4. FCR axon excitability parameters in healthy control limbs

Excitability parameter	Current study	Jankelowitz and Burke, 2009*	Percent
Stimulus-response			
CMAP peak (mV)	9.8 ± 0.3	9.6 ± 1.1	102
Stimulus (mA) for 50%max	5.9 ± 0.4	10.1 ± 1.1[+]	58
Stimulus-response slope	3.5 ± 0.1	3.0 ± 1.1	117
Stimulus width-charge			
SDTC (ms)	0.487± 0.018	0.43 ± 0.01	113
Rheobase (mA)	3.8 ± 0.3	6.9 ± 1.1	55
Recovery cycle			
RRP (ms)	3.73 ± 0.16	3.7 ± 1.0	100
Refractoriness at 2.5 ms (%)	33.1 ± 3.6	47.0 ± 4.2	70
Superexcitability (%)	-8.2 ± 1.3	-9.1 ± 1.5	90
Subexcitability (%)	13.1 ± 1.6	14.7 ± 2.2	89
TE to ± 40% currents			
TEd(10-20 ms) (%)	61.9 ± 1.0	55.9 ± 1.3	110
TEd(40-60 ms) (%)	49.7 ± 1.0	45.1 ± 0.9	110
TEd(90-100 ms) (%)	44.9 ± 1.1	41.7 ± 0.6	108
TEd(undershoot) (%)	-12.9 ± 0.6	-11.4 ± 1.0	113
S2 accommodation (%)	17.0 ± 0.7	14.6 ± 1.1	116
TEh(10-20 ms) (%)	-69.4 ± 1.1	-64.6 ± 2.0	107
TEh(20-40 ms) (%)	-85.8 ± 1.7	-81.7 ± 2.3	105
TEh(90-100 ms) (%)	-120.1 ± 3.5	-117.3 ± 3.9	102
TEh(overshoot) (%)	5.9 ± 0.6	7.2 ± 0.5	82
I/V relationship			
Resting I/V slope	0.60 ± 0.01	0.64 ± 0.00	94
Minimum I/V slope	0.18± 0.00	0.20 ± 0.00	90
Hyperpolarizing I/V slope	0.23 ± 0.01	0.3 ± 0.00	77

Values are means ± SE

* 7 males and 8 females, mean age 44.2 y. Mean values taken from their Table 1.

[+] The mean stimulus required to produce the unconditioned test CMAP (~40% of maximum).

Percent = current study mean/previous study mean x 100

Table 5. FCR axon excitability parameters in the paretic and non-paretic limbs after stroke

Excitability parameter	Paretic Current study	Paretic Huynh et al., 2013*	%	Nonparetic Current study	Nonparetic Huynh et al., 2013*	%
Stimulus-response						
CMAP peak (mV)	7.6 ± 0.7	7.6 ± 1.1	100	9.4 ± 0.4	6.4 ± 1.1	146
Stimulus (mA) for 50%max	7.9 ± 0.7	10.8 ± 1.2+	73	7.4 ± 0.6	9.4 ± 1.2	79
Stimulus width-charge						
SDTC (ms)	0.456 ± 0.015	0.47 ± 0.02	97	0.418 ± 0.016	0.45 ± 0.04	93
TE to ± 40% currents						
TEd(90-100 ms) (%)	50.4 ± 1.8	46.5 ± 3.5	108	51.0 ± 1.6	45.1 ± 3.0	113
S2 accommodation (%)	15.9 ± 0.6	15.3 ± 2.0	104	16.0 ± 1.0	15.7 ± 1.9	102
TEh(90-100 ms) (%)	-138.7 ± 7.1	-122.3 ± 3.0	113	-136.2 ± 5.5	-115.5 ± 4.3	118
I/V relationship						
Resting I/V slope	0.53 ± 0.03	0.64 ± 0.04	83	0.52 ± 0.02	0.61 ± 0.08	85
Minimum I/V slope	0.17 ± 0.00	0.17 ± 0.03	100	0.17± 0.00	0.19± 0.02	89

Values are means ± SE

* Stroke subjects (N = 5, 63 y old, 4.4 y post stroke, Fugl-Meyer score, 5.6). Mean values taken from their Table 3, before botox injection.

+ The mean stimulus required to produce the unconditioned test CMAP (~40% of maximum).

% = current study mean/previous study mean x 100.

Fit to control limb

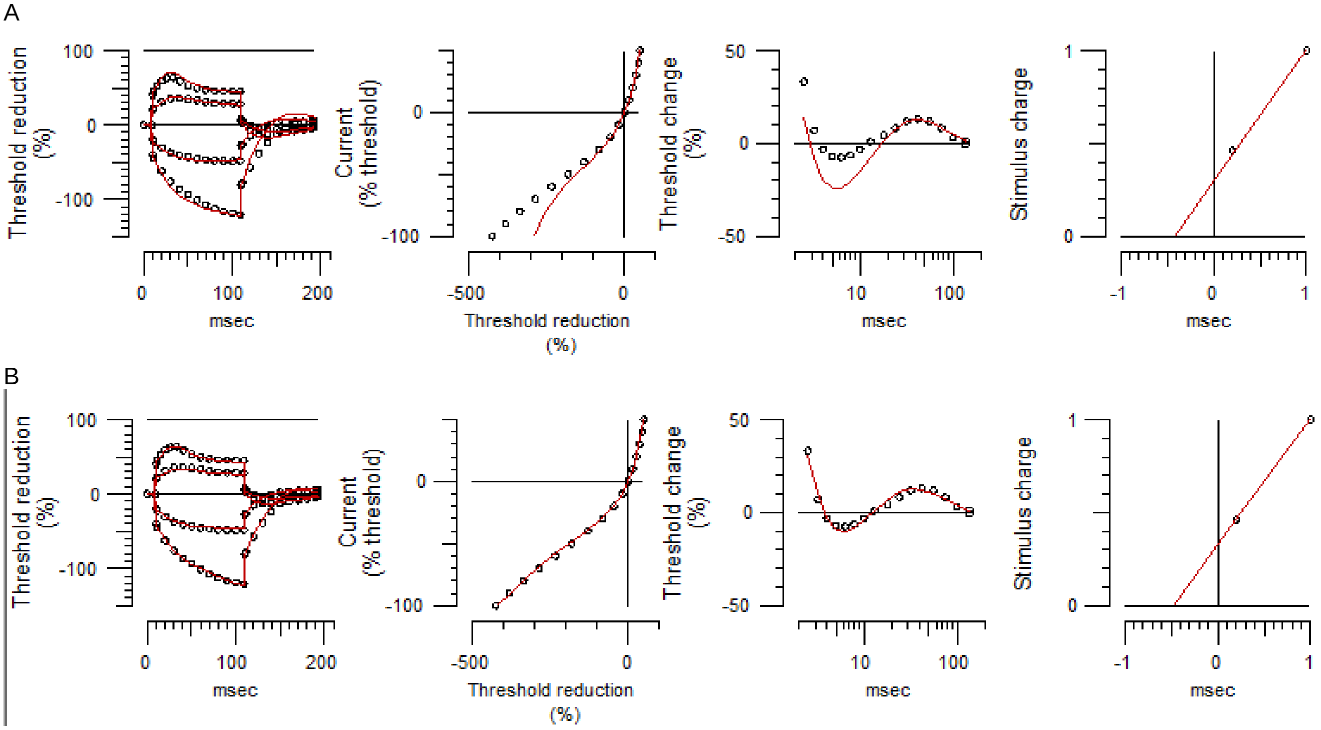

Fig 1. Modeling FCR axonal behavior in the healthy control subjects. **(A)** Control group mean FCR responses (unfilled symbols) are shown together with the best fit model for abductor pollicis brevis (APB) axonal behavior in normal controls reported previously (Kiernan et al., 2000) (red line; NC29 parameters included in the Qtrac software). Relative to APB axons, FCR axons show larger thresholds during the strongest hyperpolarizing currents of the current-threshold (I/V) test and larger refractoriness and smaller superexcitability in the recovery cycle. These FCR-APB axonal differences are consistent with the actual recorded differences in heathy adults (Jankelowitz et al., 2009). **(B)** Control group mean FCR responses (unfilled symbols) together with the best fit model, starting with the NC29 parameters (same as Fig. 3A). Changes in a number of parameters were necessary to fit the FCR responses (see text of manuscript and Supplementary Table 1). Left to right panels: Threshold electrotonus, I/V relationship, recovery cycle, and strength-duration properties.

Fit to nonparetic limb

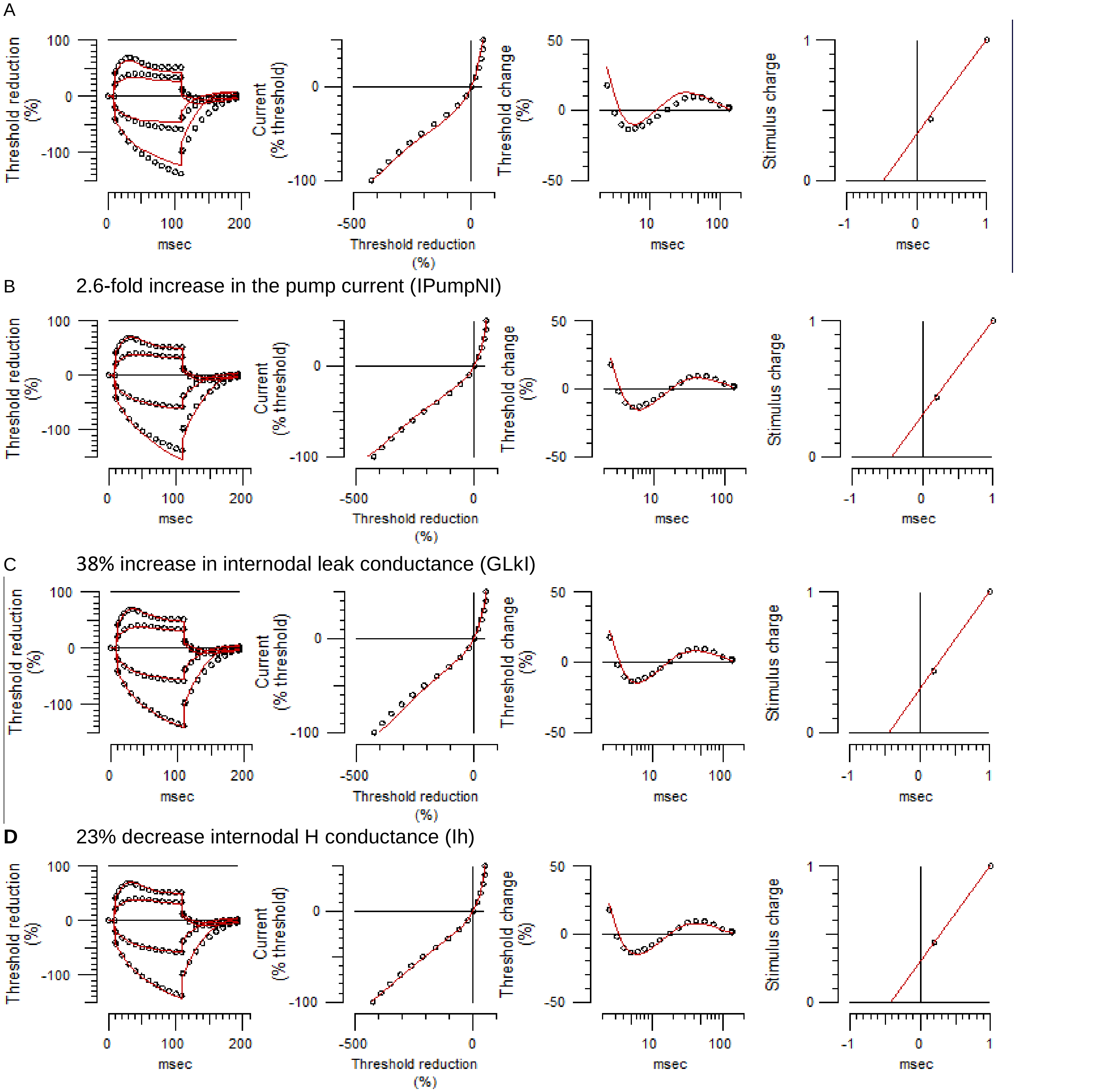

Fig. 2. Modeling nonparetic limb FCR axonal behavior. Unfilled symbols in all panels are the nonparetic limb mean recorded responses. **(A)** Nonparetic limb mean responses together with the modeled control limb responses (red line). The differences between these limbs may be explained by stroke-induced changes in axonal properties; namely, membrane hyperpolarization and altered makeup of hyperpolarization-activated (HCN) channels as suggested by the modeled responses in B-D. **(B)** A 2.6 fold increase in the pump current, from 0.0068 to 0.0175, lead to a 2.1 mV hyperpolarization of the nodal resting potential; from ~ -85.5 mV in the control group to 87.6 mV in the nonparetic limb. **(C)** A 38% increase [(3.1-2.25)/2.25 x 100] in internodal leak conductance (GLkI) was necessary to further improve the fit to hyperpolarizing threshold electrotonus (from a control group value of 2.25 to 3.1 in the nonparetic limb). **(D)** Finally, a 23% reduction in internodal H conductance (Ih) was needed to further improve the fit of the strongest hyperpolarizing currents of the I/V test (from a control group value of 1.55 to 1.2 in the nonparetic limb) (same as Fig. 3B). The net result of these changes was a 2.3 mV hyperpolarization of the nodal resting potential in the nonparetic limb (from -85.5 mV in the control group to 87.8 mV in the nonparetic limb) and a 2.4 mV hyperpolarization of the internodal resting potential (from -85.7 mV in the control group to 88.1 mV in the nonparetic limb).

Fit to the paretic limb

A

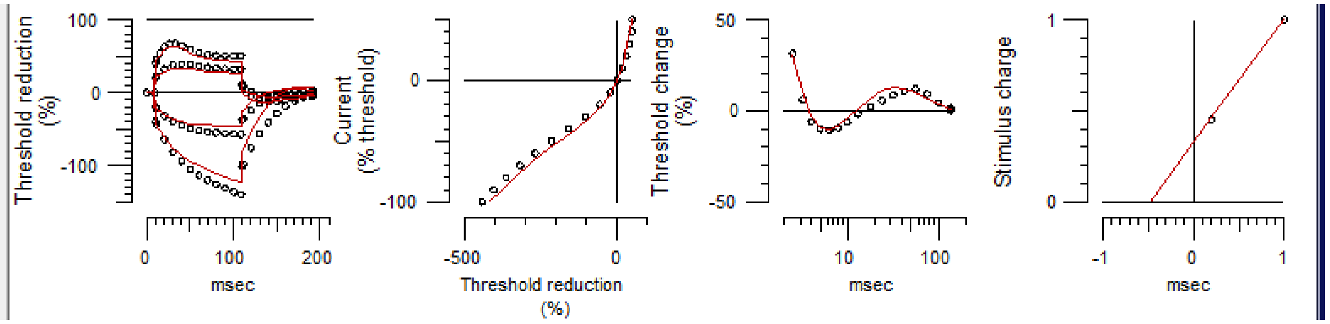

B 2.6-fold increase in the pump current (IPumpNI)

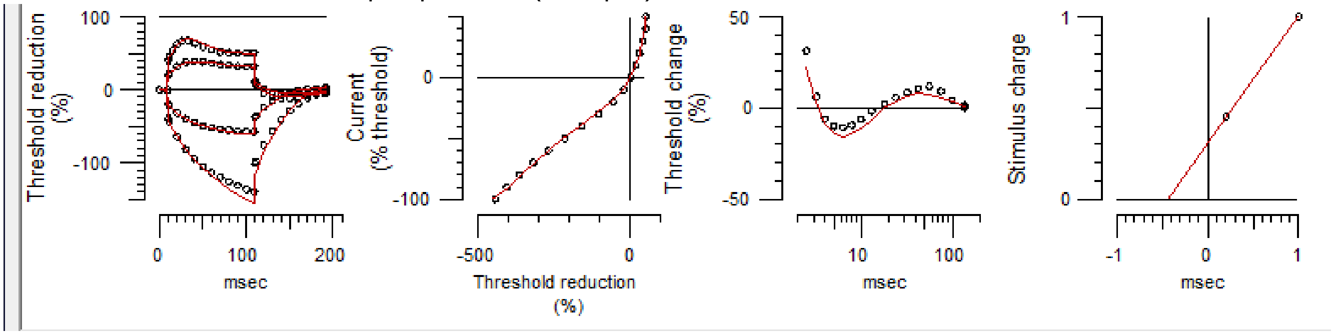

C 33% increase in internodal leak conductance (GLkI)

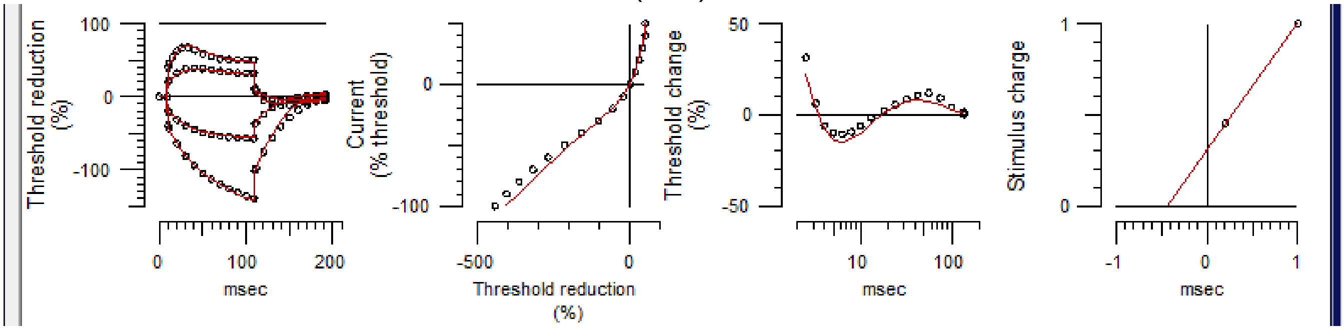

D 29% decrease in internodal H conductance (Ih)

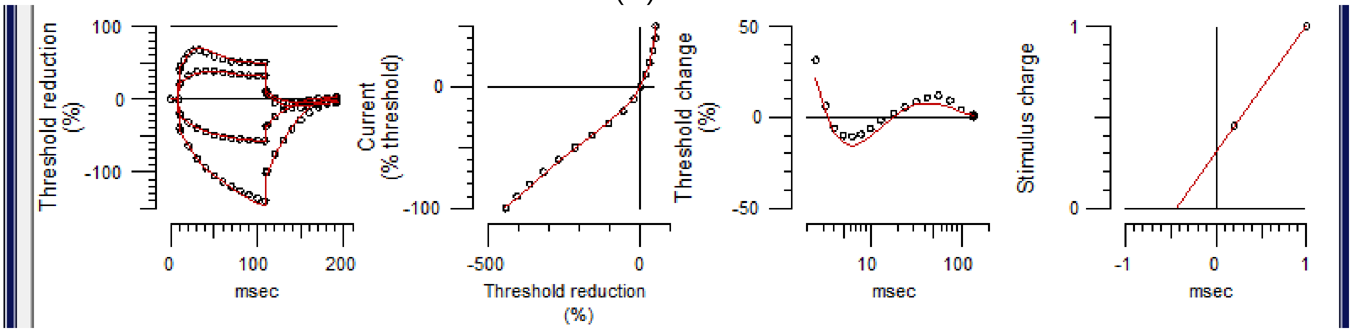

E 14% decrease in rate activation of the Na^+ channel h gate (Aah)

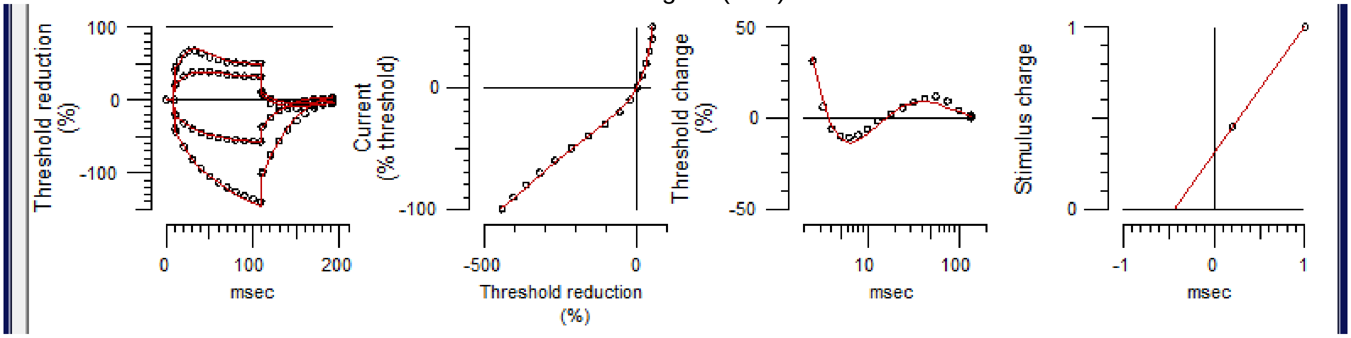

Fig. 3. Modeling paretic limb FCR axonal behavior starting with the control limb model parameters. Unfilled symbols in all panels are the paretic limb mean recorded responses. **(A)** Paretic limb mean responses together with the modeled control limb responses (red line). The differences between these limbs may be explained by stroke-induced changes in axonal properties; namely, membrane hyperpolarization, altered makeup of HCN channels, and altered Na^+ channel gating as suggested by the modeled responses in B-E. **(B)** A 2.6 fold increase in the pump current, from 0.0068 to 0.0175, lead to a 2.1 mV hyperpolarization of the nodal resting potential; from ~ -85.5 mV in the control group to 87.6 mV in the paretic limb. **(C)** A 33% increase in internodal leak conductance (GLkI) was necessary to further improve the fit to hyperpolarizing threshold electrotonus (from a control group value of 2.25 to 3.0 in the paretic limb). **(D)** A 29% reduction in internodal H conductance (Ih) was needed to further improve the fit of the strongest hyperpolarizing currents of the I/V test (from a control group value of 1.55 to 1.1 in the paretic limb). **(E)** A 14% reduction in the rate activation of the Na^+ channel h gate (Aah) was needed to better fit the refractory period and superexcitability: from 0.0245 in the control limb to 0.021 in the paretic limb (same as Fig. 3C). The net result of these changes was a 2.3 mV hyperpolarization of the nodal resting potential in the paretic limb (from -85.5 mV in the control group to 87.8 mV in the paretic limb) and a 2.5 mV hyperpolarization of the internodal resting potential (from -85.7 mV in the control group to 88.2 mV in the paretic limb).

Fit to the paretic limb

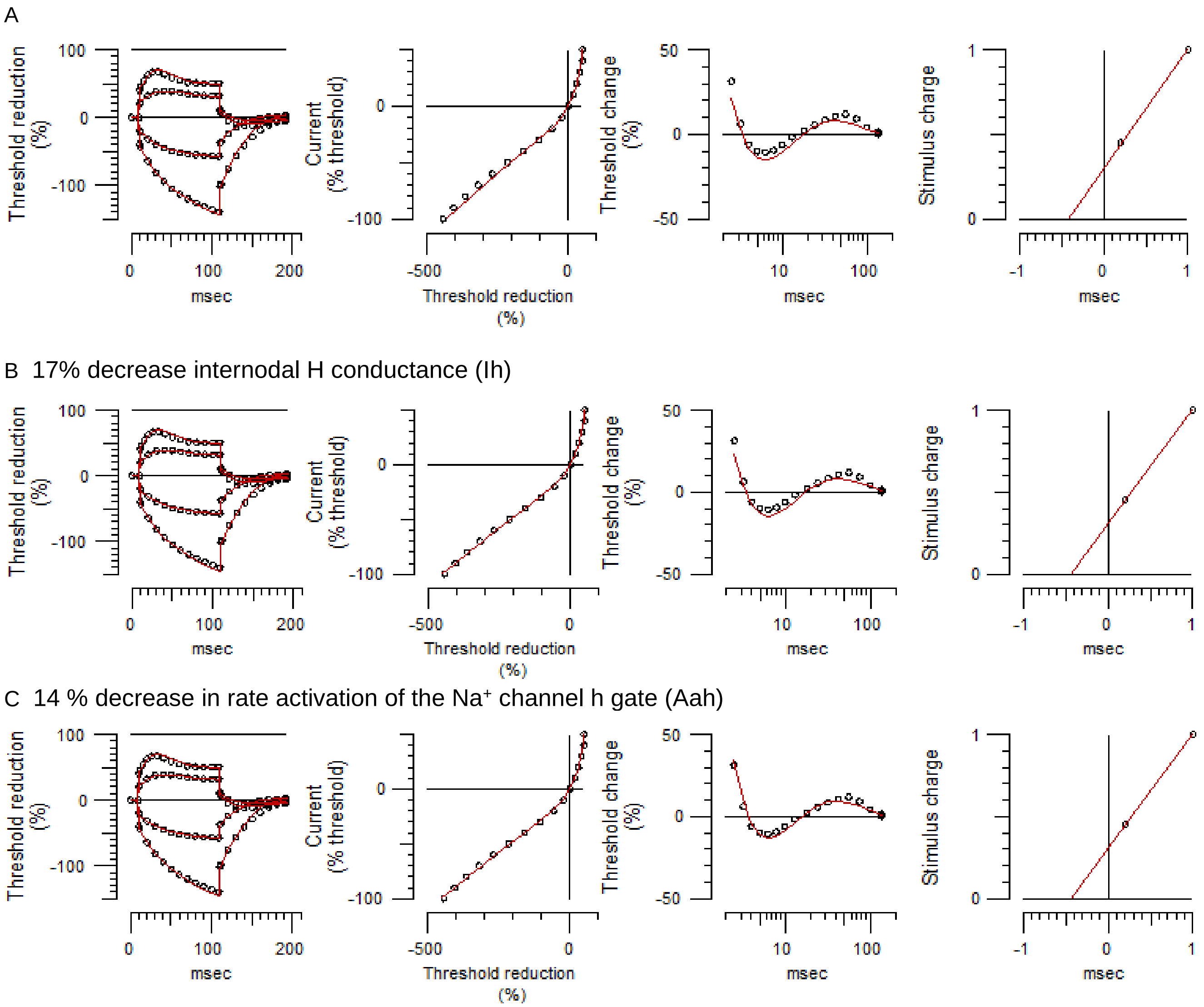

Fig. 4. Modeling paretic limb FCR axonal behavior starting with the nonparetic limb model parameters. Unfilled symbols in all panels are the paretic limb mean recorded responses **(A)** Paretic limb mean responses together with the modeled nonparetic limb responses (red line). The subtle differences between limbs, apparent during hyperpolarizing responses of the I/V test and during the recovery cycle, may be explained by differences in ion channel properties as suggested by the modeled responses in B-C. **(B)** A 17% reduction in H conductance (Ih) was needed to best fit the stronger hyperpolarizing I/V responses; from 1.2 in the nonparetic limb to 1.0 in the paretic limb**. (C)** A 14% reduction in the rate activation of the Na^+ channel h gate (Aah) was needed to better fit the refractory period and superexcitability: from 0.0245 in the nonparetic limb to 0.021 in the paretic limb (same as Fig. 3D).

References

Huynh W, Krishnan AV, Lin CS, Vucic S, Katrak P, Hornberger M, et al. Botulinum toxin modulates cortical maladaptation in post-stroke spasticity. Muscle Nerve 2013;48:93-9.

Jankelowitz SK, Burke D. Axonal excitability in the forearm: normal data and differences along the median nerve. Clin Neurophysiol 2009;120:167-73.

Kiernan MC, Burke D, Andersen KV, Bostock H. Multiple measures of axonal excitability: a new approach in clinical testing. Muscle Nerve 2000;23:399-409.
